# Family Caregivers’ Needs for Integrating Home Care Technology and their Everyday Caregiving

**DOI:** 10.1093/geroni/igaf122.2180

**Published:** 2025-12-31

**Authors:** Tomoko Wakui, Sakiko Itoh, Hiroyasu Miwa, Kentaro Watanabe

**Affiliations:** Tokyo Metropolitan Institute for Geriatrics and Gerontology, Tokyo, Tokyo, Japan; Institute of Science Tokyo, Tokyo, Tokyo, Japan; National Institute of Advanced Industrial Science and Technology, Kashiwa, Chiba, Japan; National Institute of Advanced Industrial Science and Technology, Kashiwa, Chiba, Japan

## Abstract

Beyond addressing care recipients’ needs, family caregiving factors also play a crucial role in the adoption of home care technologies. This study explores the factors influencing family caregivers’ preferences for integrating such technologies into daily caregiving. We surveyed 2,002 family caregivers of community-dwelling older adults, selected based on the national percentage of older adults requiring long-term care under Japan’s public long-term care insurance program. The primary outcome was caregivers’ preferences for 17 types of home care technologies, such as bathing aids, toileting prediction, mobility support, medication management, and GPS tracking. We controlled for care recipient characteristics (age, dementia status, ADLs), and examined family caregiver characteristics (age, marital/employment status, income, health), and caregiving situations (frequency, duration, co-residence, burden). Logistic regression analyses revealed that higher caregiving burden was significantly associated with preferences for bathing aids (OR: 1.04, p < .01), toileting prediction (OR: 1.03, p < .01), GPS trackers (OR: 1.03, p = .004), and shopping assistance tools (OR: 1.03, p = .01). Live-out caregivers were more likely to prefer communication robots (OR: 1.47, p = .03). Higher household income was linked to preferences for bathing aids, toileting support, pill dispensers, fall detection monitors, and social robots (p < .05). Our findings highlight that family caregivers’ circumstances—not just care recipients’ needs—significantly shape technology adoption preferences, emphasizing the need for caregiver-centered solutions.

